# Matrine combined with cisplatin synergistically inhibited urothelial bladder cancer cells via down-regulating VEGF/PI3K/Akt signaling pathway

**DOI:** 10.1186/s12935-017-0495-6

**Published:** 2017-12-28

**Authors:** Xiao-Zhong Liao, Lan-Ting Tao, Jia-Hui Liu, Yue-Yu Gu, Jun Xie, Yuling Chen, Mei-Gui Lin, Tao-Li Liu, Dong-Mei Wang, Hai-Yan Guo, Sui-Lin Mo

**Affiliations:** 1 The First Affiliated Hospital, Sun Yat-sen University, Guangzhou, 510080 People’s Republic of China; 2 The Second Clinical College, Guangzhou University of Chinese Medicine and Guangdong Provincial Academy of Chinese Medical Sciences, Guangzhou, 510120 People’s Republic of China; 3 Peking Union Medical College Hospital, Peking Union Medical College, China Academy of Medical Sciences, Beijing, 100730 People’s Republic of China; 4 Sydney Acupuncture & Chinese Medicine Centre, Hurstville, NSW 2220 Australia; 5 Liwan District Shi wei tang Street Community Health Service Center, Guangzhou, 510360 People’s Republic of China; 6 The Seventh Affiliated Hospital, Sun Yat-sen University, Shenzhen, 518107 People’s Republic of China; 7 School of Pharmaceutical Sciences, Sun Yat-sen University, Guangzhou, 510006 People’s Republic of China; 8 Graduate School of China Academy of Chinese Medical Sciences, Beijing, 100700 People’s Republic of China

**Keywords:** Matrine, Cisplatin, Combination, Synergistic effect, EJ, T24, VEGF/PI3K/Akt signal pathway

## Abstract

**Background:**

Cisplatin is one of the first-line drugs for urothelial bladder cancer (UBC) treatment. However, its considerable side effects and the emergence of drug resistance are becoming major limitations for its application. This study aimed to investigate whether matrine and cisplatin could present a synergistic anti-tumor effect on UBC cells.

**Methods:**

Cell viability assay was used to assess the suppressive effect of matrine and cisplatin on the proliferation of the UBC cells. Wound healing assay and transwell assay were applied respectively to determine the migration and invasion ability of the cells. The distribution of cell cycles, the generation of reactive oxygen species (ROS) and the apoptosis rate were detected by flow cytometry (FCM). The expressions of the relative proteins in apoptotic signal pathways and the epithelial–mesenchymal transition (EMT) related genes were surveyed by western blotting. The binding modes of the drugs within the proteins were detected by CDOCKER module in DS 2.5.

**Results:**

Both matrine and cisplatin could inhibit the growth of the UBC cells in a time- and dose-dependent manner. When matrine combined with cisplatin at the ratio of 2000:1, they presented a synergistic inhibitory effect on the UBC cells. The combinative treatment could impair cell migration and invasion ability, arrest cell cycle in the G1 and S phases, increase the level of ROS, and induce apoptosis in EJ and T24 cells in a synergistic way. In all the treated groups, the expressions of E-cadherin, β-catenin, Bax, and Cleaved Caspase-3 were up-regulated, while the expressions of Fibronectin, Vimentin, Bcl-2, Caspase-3, p-Akt, p-PI3K, VEGFR2, and VEGF proteins were down-regulated, and among them, the combination of matrine and cisplatin showed the most significant difference. Molecular docking algorithms predicted that matrine and cisplatin could be docked into the same active sites and interact with different residues within the tested proteins.

**Conclusions:**

Our results suggested that the combination of matrine and cisplatin could synergistically inhibit the UBC cells’ proliferation through down-regulating VEGF/PI3K/Akt signaling pathway, indicating that matrine may serve as a new option in the combinative therapy in the treatment of UBC.

## Background

Urothelial bladder cancer (UBC) is the 7th most common tumor worldwide in male and the 17th in females, and is one of the most fatal urothelial malignancies. Although almost three quarters of recently diagnosed UBCs are still not invasive [1], it is estimated that 50–70% of newly diagnosed bladder cancers will recur and 10–30% will progress to muscle-invasive carcinoma [2]. Once bladder cancer progresses into the invasive stage, the prognosis becomes poor [3], thus, the treatment depends on the stage of the cancer. It may include several combinations of surgery, radiation therapy, chemotherapy, or immunotherapy [2]. Currently the standard first-line treatment for UBC is cisplatin-based chemotherapy.

Cisplatin, one of the most famous chemotherapeutic drugs, has been applied for the treatment of many human cancers, including bladder, lung, head and neck, ovarian, and testicular cancers. However, the major limitations for its application were firstly its drug resistance, which was potentially caused by changes in cellular uptake and efflux of cisplatin, increasing biotransformation and detoxification in the liver, and increasing DNA repair and anti-apoptotic mechanisms, as well as its considerable side effects, e.g. severe kidney problems, allergic reactions, decrease immunity to infections, gastrointestinal disorders, hemorrhages, and hearing loss. Therefore, the combinative treatments of cisplatin and other anticarcinogens have caused concern for withstanding the drug-resistance, weakening the toxicity, and increasing the chemotherapeutic efficacy [4].

**Fig. 1:**
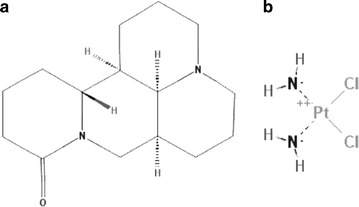
The 2D structure of matrine (**a**) and cisplatin (**b**) (from PubChem compound http://pubchem.ncbi.nlm.nih.gov/)

Matrine is a primary active ingredient from the dry roots of *Sophora flavescens*, named ‘Ku-Shen’ in traditional Chinese medicine [5]. Its molecular formula is C_15_H_24_N_2_O with its molecular mass of 248.37 g/mol and its compound ID (CID) in PubChem Compound of 91466 (Fig. 1).

It has been confirmed that matrine could suppress the proliferation of numerous cancer cells in vitro, such as lung cancer [6], gastric cancer [7], liver cancer [8], breast cancer [9] and bladder cancer [10] cells. However, the anti-cancer effect of matrine is too weak to be used in antineoplastic chemotherapy alone. It has been documented that matrine could lead to apoptosis in NSCLC cells that were cisplatin-resistant [11]. Additionally, it has been found that the combination of matrine with cisplatin could obviously improve the antineoplastic effect on U14 rats with cervical cancer [12]. It is believed that matrine might serve as a new option in the combinative therapy in the treatment of UBC. However, the inhibition of matrine combined with cisplatin on the proliferation of UBC cells and the underlying molecular mechanisms remain unclear.

Thus, in the study, we explored whether matrine and cisplatin could present a synergistic anti-tumor effect on the UBC cells and the potential molecular mechanisms based on the platform built up in our previous studies for assessing the combinative effects of drugs and interactions between drugs and tested proteins [13].

## Methods

### Cell lines, culture condition, and reagents

The UBC cell lines EJ, T24, BIU, 5637 were gifts from the State Key Laboratory of Oncology in South China. All cell lines were cultured in RPMI 1640 medium (Invitrogen Corp, Carlsbad, CA, USA) supplemented with 10% fetal bovine serum (Invitrogen Corp, Carlsbad, CA, USA), 100 U/mL penicillin and 100 U/mL streptomycin (Gino, Hangzhou, Zhejiang, China) in a humidified incubator at 37 °C with 5% CO_2_. Matrine and cisplatin were purchased from Sigma (St. Louis, MO, USA). Matrine was dissolved in physiological saline to make a 100 mM stock solution and stored at − 20 °C for future use. Cisplatin was dissolved in physiological saline to make a 10 mM stock solution and stored at − 20 °C for future use.

### Cell viability assay

The anti-proliferative effects of matrine and cisplatin on UBC cell lines were detected by CCK-8 kit (Dojindo Laboratories, 119 Kumamoto, Japan). EJ, T24, BIU, or 5637 cells (8.0 × 10^3^ cells each well) were seeded into 96-well plates. The cells were cultured with various concentrations of matrine and cisplatin respectively and concurrently. After treatment for 24, 48 or 72 h, cells were incubated for additional 90 min with 10 μL of CCK-8 solution. Finally, the optical density was measured at 450 nm by the microculture plate reader (Thermo Scientific, Rockford, IL, USA). The proliferative inhibition rate was calculated using the formula: proliferative inhibition rate = (1 − experimental group/control group) × 100%. The 50% inhibitory concentration (IC_50_) value was calculated by nonlinear regression analysis using SPSS20.0 software.

### Synergy determination

The combination index (CI) was determined by the isobologram analysis for the combination study which was based upon the Chou–Talalay method. The data obtained from the cell viability assay were standardized to the control group and showed as % viability. Further, the data was converted to fraction affected (Fa; range 0–1; where Fa = 0 represents 100% viability and Fa = 1 represents 0% viability) and analyzed by the CompuSyn™ program (Biosoft, Ferguson, MO) based upon the Chou–Talalay method. The CI values represent the modes of the interaction between two drugs. The CI < 1 indicates synergism, CI = 1 indicates an additive effect and CI > 1 indicates antagonism.

### Wound healing assay

EJ and T24 cells (1 × 10^6^/1 mL/well) in logarithmic phase were plated into 6-well plates. After 24 h, the adhesive cells were scratched along a straight line using a 200 μL pipette tip, then the scraped cells and cell debris were cleared with PBS for three times. Fresh serum free medium including various drugs were added to 6-well plates, and the cells were allowed to repair the scratches for 24 h. Pictures (magnification, 10×) were taken at 0 and 24 h at the same place where was scratched. And then, Adobe Photoshop CS6 software was applied to measure the moving distance of cells.

### Transwell assay

The transwell filters (8 μm pore, 6.5 mm polycarbonate, Corning, NY, USA) were coated with a thin layer of Matrigel Basement Membrane Matrix (BD Biosciences, Bedford, MA). EJ and T24 cells (3 × 10^4^) in logarithmic phase were resuspended in 500 μL serum-free medium containing different drug treatments and plated on the above compartment, and 800 μL full medium including 10% FBS was added to the nether compartment. The transwell filters were put in a humidified incubator at 37 °C with 5% CO_2_ for 24 h. Afterwards, the cells attached to the lower surface of membrane were fixed with 4% paraformaldehyde at room temperature for 30 min and stained with 0.5% crystal violet. The cells on the upper surface of the filter were removed by wiping with a cotton swab. Then the number of stained cells on the lower surface was counted using the microscope (magnification, 100×). A total of 5 fields were counted for each transwell filter.

### Flow cytometric cell cycle analysis

The cell cycle detection kit purchased from 4A Biotech Co., Ltd. was used to detect the cell cycle. EJ and T24 cells (5.0 × 10^5^/1 mL/well) in logarithmic phase were plated into 6-well plates with full medium containing different drug treatments in a humidified incubator at 37 °C with 5% CO_2_ for 48 h. The treated cells were collected and then washed with cold PBS. Subsequently, 70% cold ethanol was applied to immobilize the harvested cells at 4 °C overnight. The cells were washed with cold PBS again and incubated with 100 μL RNase at 37 °C water-bath water for 30 min, and then labeled with 400 μL propidium iodide (PI) and incubated for 30 min at room temperature in the dark. For each detection, 50,000 cells were tested at least. ACEC NovoCyte flow cytometer equipped with Novoexpress (Becton–Dickinson, San Jose, CA, USA) was applied to detect the cell cycle.

### Flow cytometric reactive oxygen species analysis

The reactive oxygen species assay kit purchased from 4A Biotech Co., Ltd. was used to measure the level of reactive oxygen species (ROS) in treated cells. EJ and T24 cells (5.0 × 10^5^/1 mL/well) in logarithmic phase were plated into 6-well plates and treated with different drugs for 48 h. The treated cells were collected and then washed with cold PBS. Subsequently, cells were stained with DCFH-DA for 30 min at room temperature in the dark. The levels of ROS in treated cells were measured immediately after staining by using ACEC NovoCyte flow cytometer equipped with Novoexpress (Becton–Dickinson, San Jose, CA, USA).

### Apoptosis assay

The Annexin V-FITC apoptosis detection kit purchased from 4A Biotech Co., Ltd. was applied to detect cell apoptosis. EJ and T24 cells (5.0 × 10^5^/1 mL/well) in logarithmic phase were plated into 6-well plates and treated with different drugs for 48 h. The treated cells were collected and washed with cold PBS after treatment. In accordance with the manufacturer’s instructions, the cells were stained with Annexin V-FITC and PI for 30 min at room temperature in the dark, the apoptosis rate of treated cells were determined immediately after staining by using ACEC NovoCyte flow cytometer equipped with Novoexpress (Becton–Dickinson, San Jose, CA, USA).

### Western blot analysis

EJ and T24 cells in logarithmic phase were treated with different drugs for 48 h. After treatment, the EJ, T24 cells were harvested and lysed with lysis buffer. The cell lysates were incubated on ice for 30 min and then centrifuged at 12,000*g* for 10 min at 4 °C, the supernatants were collected and determined by BCA protein assay kit (Beyotime, Jiangsu, China). All the selected protein extracts of tested cells were resolved by SDS-PAGE and transferred to polyvinylidenedifluoride (PVDF) membranes (0.22 μm, Millipore, MA, USA). After blocking for 1 h in 5% skim milk, the PVDF membranes incubated overnight at 4 °C with primary antibodies (E-cadherin 1:1000, β-catenin 1:1000, Fibronectin 1:1000, Vimentin 1:1000, VEGF 1:1000, VEGFR2 1:1000, PI3K 1:1000, p-PI3K 1:1000, p-Akt 1:1000, Akt1:1000, Cleaved Caspase-3 1:500, caspase-3 1:500, Bcl-2 1:500, Bax 1:500 and GAPDH 1:8000). All the primary antibodies were obtained from Cell Signaling Technology (Danvers, MA, USA). Following washed with Tris buffered saline including 0.1% Tween-20 (TBST) for three times, membranes were incubated with horseradish peroxidase (HRP)-conjugated secondary antibodies (Cell Signaling Technology, Danvers, MA, USA) at room temperature for 1 h. After washing with TBST again, immunoreactivity of the membranes were detected using the Bio-Rad-Image-Lab with an electrochemiluminescence system (ECL) (Thermo Fisher Scientific, MA, USA). The densitometry of the protein bands were measured using the ImageJ (NIH image software) and normalized to their relevant controls.

### Molecular docking

To understand the potential interactions between the tested drugs and the selected proteins, molecular docking algorithm was carried out in this study with Discovery Studio (DS) 2.5. The two dimensional (2D) structures of matrine and cisplatin were found in the database of PubChem (http://pubchem.ncbi.nlm.nih.gov/), with the PubChem CID of 91466 and 441203 respectively. The 3D structure of PI3K (PDB-ID: 4J6I), AKT2 (PDB-ID: 2JDR), Caspase-3 (PDB-ID: 2XYH), Bcl-2 (PDB-ID: 4IEH), the targeted proteins, could be acquired from the database of Protein Data Bank (PDB http://www.rcsb.org/pdb/home/home.do). The procedures of virtual docking with DS 2.5 were as follows: firstly, the water molecules in the tested proteins were removed and refined with CHARMM on the targeted proteins and the selected ligands. Secondly, the possible active sites of tested proteins based on endogenous ligands were automatically found out with the algorithm. Thirdly, the drugs, and the selected ligands, were docked into the binding pocket of the tested proteins. And then, the docking model of the drugs and the tested proteins was examined by the module. Before performing the procedure, the calculation of root mean square deviation (RMSD) was carried out as the verification for the selection of the two modules (CDOCKER and Libdock) in DS 2.5.

### Statistical analysis

All experiments were repeated at least for three times. Data were showed as mean ± SD, and analyzed using GraphPad Prism 6.02 software (San Diego, CA, USA), except the IC_50_ values which were calculated by SPSS 20.0 software. Differences between groups were analyzed by the Student’s t test. A P value of 0.05 or less was considered as significant.

## Results

### Co-treatment of matrine and cisplatin synergistically inhibited the proliferation of the UBC cells

**Fig. 2:**
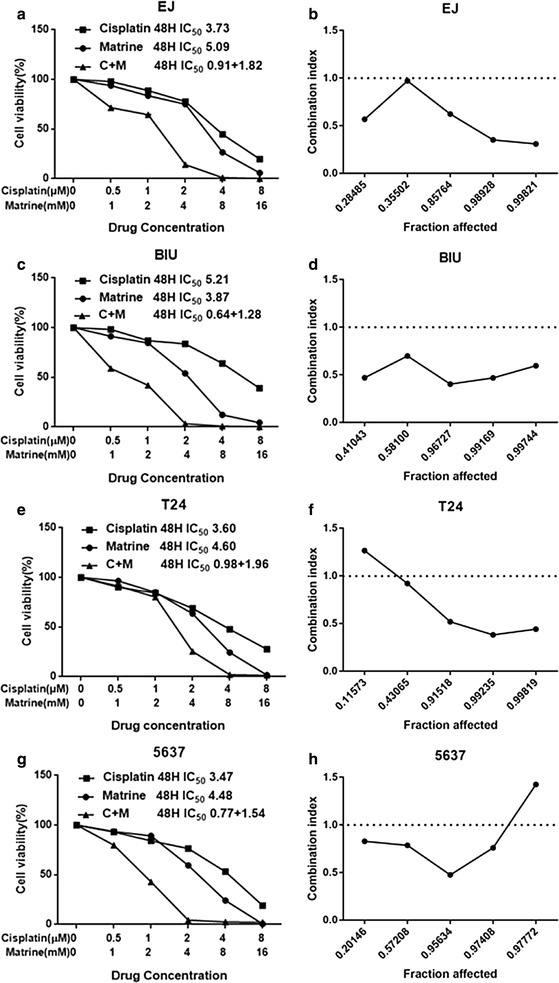
The proliferative inhibition effect of matrine, cisplatin and the combination on the UBC cells. Drug concentration-cell viability curves were drawn as the viable cell percentage based on the cell viability assay (**a**, **c**, **e**, **g**). The synergistic effects between matrine and cisplatin were exhibited as Fa-CI plots (**b**, **d**, **f**, **h**). Data are from three repeated experiments with quadruplicate wells (mean ± SD). *P < 0.05, **P < 0.01, or ***P < 0.001 versus the control group

Both matrine and cisplatin inhibited the proliferation of the UBC cells in a time- and dose-dependent manner. After 48 h treatment, the IC_50_ values of matrine for EJ, T24, BIU and 5637 cell lines were 5.09, 4.60, 3.87 and 4.48 mM respectively, those of cisplatin were 3.73, 3.60, 5.21 and 3.47 μM respectively (Fig. 2).

In accordance with the IC_50_ of matrine and cisplatin, we set up the combination group at the fixed molar ratio of 1:2000 (cisplatin:matrine) for 48 h treatment. Compared with the individual drug, the drug combination produced a stronger suppressive effect on cells proliferation. The combination of matrine with cisplatin showed a synergistic inhibitory effect on T24 cells when Fa value was ≥ 0.43 and 5637 cells when Fa value was ≤ 0.97. The synergism of drug combination treatment was observed in EJ and BIU cells, whatever the Fa value was. The summary of CI and the concentration of separate drugs in combination at 50% Fa were shown in Table 1.

**Table 1:** Summary of CI value and the concentration of separate drugs in combination at 50% Fa

Drug combination	Fa = 0.5			
	EJ	T24	BIU	5637
Cisplatin + matrine				
CI regimen	0.66585 ± 0.09929	0.66555 ± 0.02893	0.676608 ± 0.13417	0.51106 ± 0.06219
Cisplatin (μM)	0.91083	0.97855	0.63823	0.76946
Matrine (mM)	1.82166	1.95910	1.27646	1.53892

### Co-treatment of matrine and cisplatin synergistically inhibited migration and invasion of the UBC cells

**Fig. 3:**
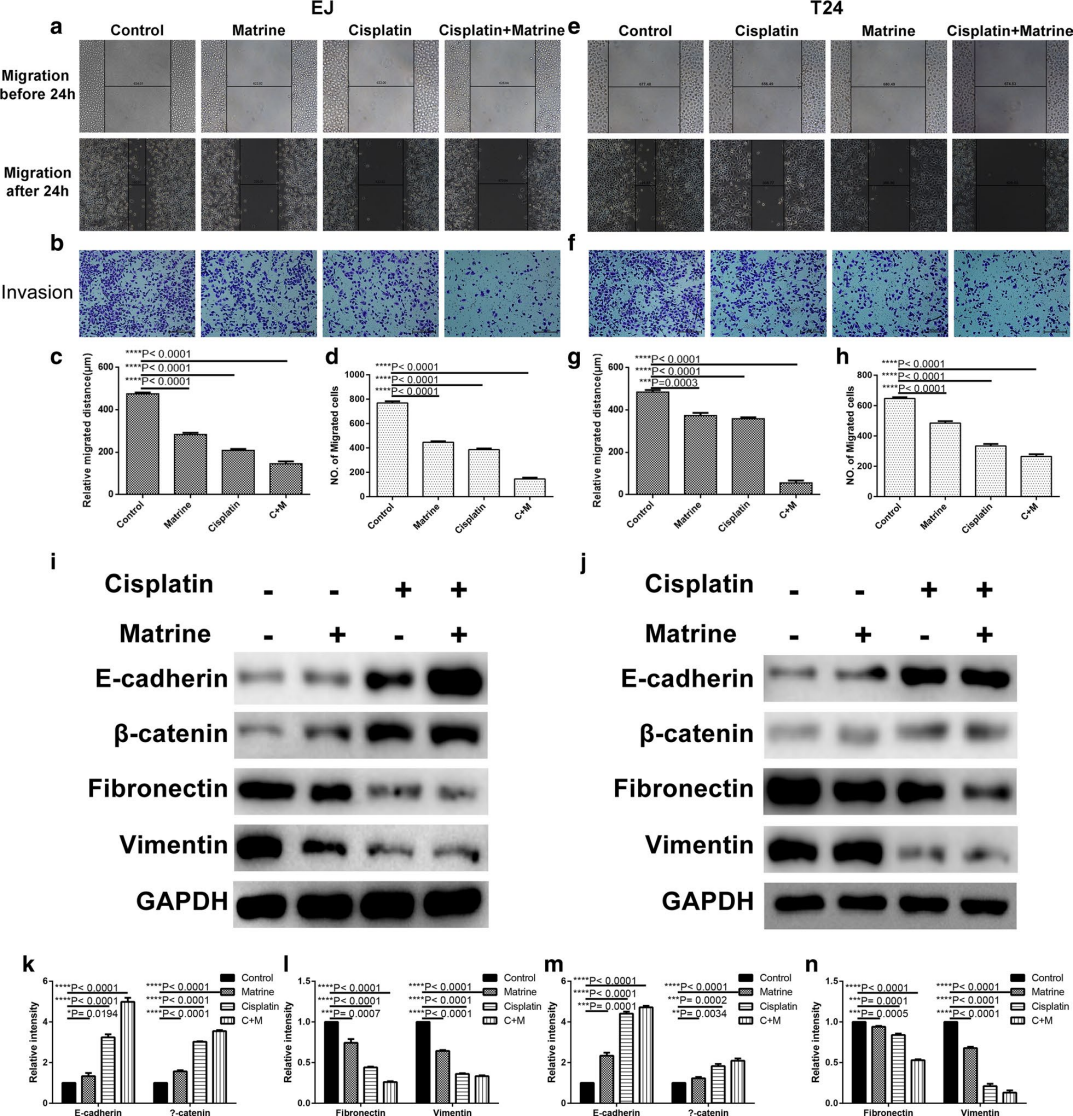
Matrine and cisplatin suppressed migration and invasion ability of EJ and T24 cells. Typical images of wound healing (**a**, **e**) and transwell assay (**b**, **f**) after 24 h treatment with 5 mM of matrine, 4 μM of cisplatin alone and 1 μM of cisplatin and 2 mM of matrine in combination. Histograms show the average migrated distance (**c**, **g**) and the amount of invasive cells (**d**, **h**), respectively. The protein expression levels of E-cadherin, β-catenin, Fibronectin, Vimentin and GAPDH of EJ and T24 cells after 24 h treatment with 4 μM of cisplatin, 5 mM of matrine and 1 μM of cisplatin and 2 mM of matrine in combination (**i**, **j**). Histograms present the relative grey value of the related proteins measured by ImageJ (**k**, **l**, **m**, **n**). All data are shown as the mean ± SD of three independent experiments. *P < 0.05, **P < 0.01, ***P < 0.001, or ****P < 0.0001 versus the control group (magnification, ×100; scale bars, 100 μm)

To identify the combination of matrine and cisplatin that achieved maximal biological function, we applied the wound healing assay and transwell assay to investigate the migration and invasion ability of EJ and T24 cells. Figure 3 showed that the migration distances and the invasive cell numbers were significantly decreased after 24 h drug treatment. Meanwhile, the combination treatment showed the least migration distance and invasive cell number. Furthermore, we performed the western blotting analysis to investigate the epithelial–mesenchymal transition (EMT) related genes (E-cadherin, β-catenin, Fibronectin, Vimentin), and it showed that after the single drug treatment in EJ and T24 cells, the expression of the epithelial markers E-cadherin and β-catenin increased, whereas the expression of the mesenchymal markers Fibronectin and Vimentin decreased, and the drug combination treatment showed the most significant difference. These findings indicated that the combination treatment of cisplatine and matrine influenced the expression of EMT related genes.

### Co-treatment of matrine and cisplatin synergistically arrested cell cycles of the UBC cells

**Fig. 4:**
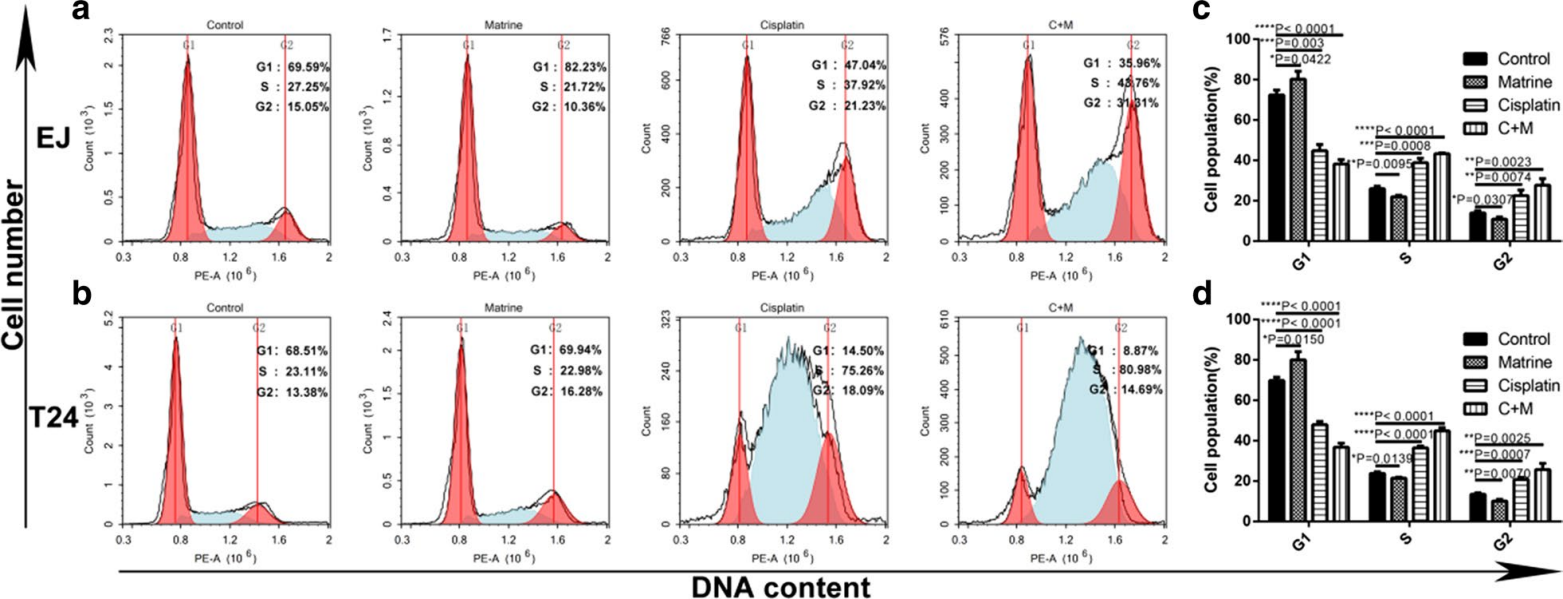
Effect of matrine and cisplatin alone and in combination on cell cycle arrest. The percentage of cells in G1, S or G2/M phase in EJ (**a**) or T24 cells (**b**) treated with 5 mM of matrine, 4 μM of cisplatin alone and 1 μM of cisplatin and 2 mM of matrine in combination for 48 h. Data represent the cell population of cell cycle arrest of EJ (**c**) and T24 (**d**). All data are shown as the mean ± SD of three independent experiments. *P < 0.05, **P < 0.01, ***P < 0.001, or ****P < 0.0001 versus the control group

After verifying the anti-proliferation effect of matrine and cisplatin, we applied FCM to analyze the cell cycle phases of the treated UBC cells. As shown in Fig. 4, matrine increased the G1 phase cell population primarily, while the cisplatin and the combination treatment increased primarily the S phase cell percentage in comparison with the untreated cells, and the latter is more significant.

### Co-treatment of matrine and cisplatin synergistically induced ROS production of the UBC cells

**Fig. 5:**
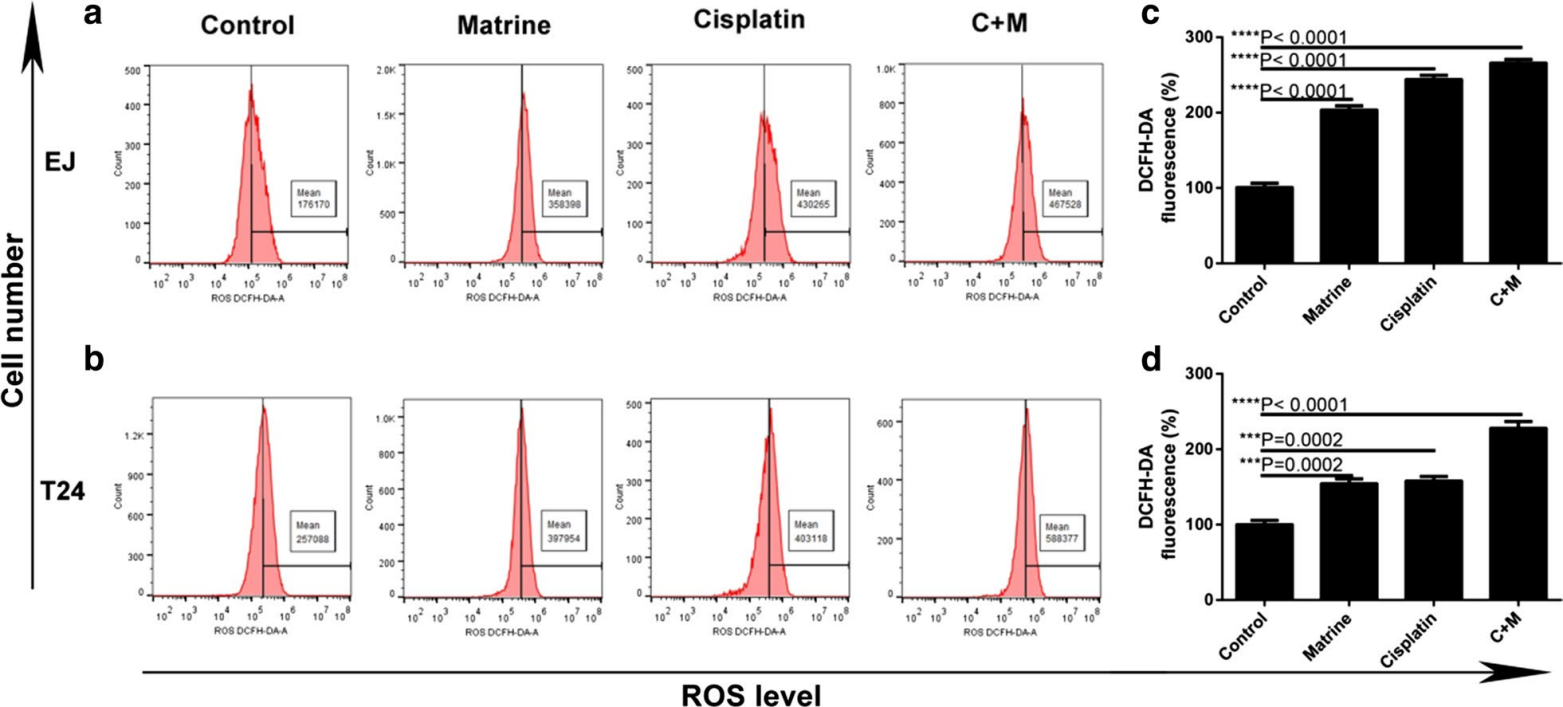
Effect of matrine and cisplatin alone and in combination on ROS level. The ROS level of EJ (**a**) and T24 cells (**b**) treated with 5 mM of matrine, 4 μM of cisplatin alone and 1 μM of cisplatin and 2 mM of matrine in combination for 48 h. Histograms represent the average ROS level of EJ (**c**) and T24 (**d**). All data are shown as the mean ± SD of three independent experiments. *P < 0.05, **P < 0.01, ***P < 0.001, or ****P < 0.0001 versus the control group

Analysis of ROS of treated cells revealed that both single drug treatment and drug combination increased the generation of ROS in EJ and T24 cells. Furthermore, the combination treatment more potently induced the generation of ROS than single treatment in EJ and T24 cells (Fig. 5).

### Co-treatment of matrine and cisplatin synergistically induced apoptosis of the UBC cells

**Fig. 6:**
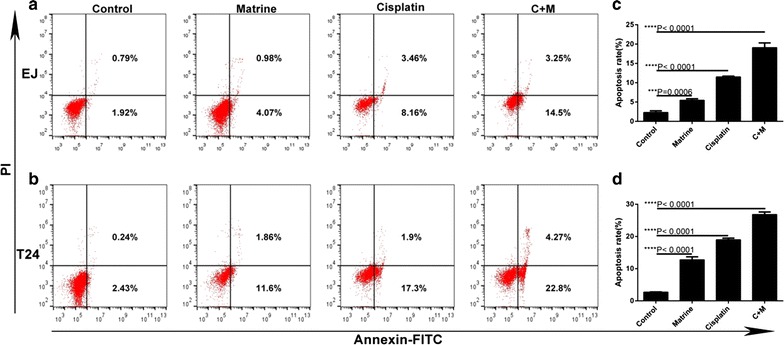
Effect of matrine and cisplatin alone and in combination on apoptosis. Representative profiles showing apoptosis in EJ (**a**) and T24 (**b**) cells treated with 5 mM of matrine, 4 μM of cisplatin alone and 1 μM of cisplatin and 2 mM of matrine in combination for 48 h. Histograms represent the average apoptosis rate of EJ (**c**) and T24 (**d**). All data are shown as the mean ± SD of three independent experiments. *P < 0.05, **P < 0.01, ***P < 0.001, or ****P < 0.0001 versus the control group

Both single drug treatment and drug combination increased the proportion of early and late apoptosis in EJ and T24 cells. Furthermore, co-treatment more potently induced apoptosis compared with single treatment in EJ and T24 cells (Fig. 6).

### Co-treatment of cisplatin and matrine synergistically decreased the activity of VEGF/PI3K/Akt signaling pathway in the UBC cells

**Fig. 7:**
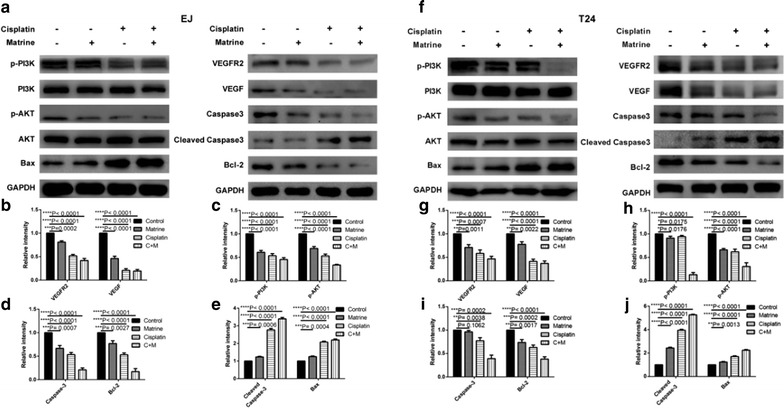
Suppressive effect of matrine, cisplatin and the combination on VEGF/PI3K/Akt signaling pathway in EJ and T24 cells. Figures are the protein expression levels of Bax, Bcl-2, Cleaved Caspase-3, Caspase-3, p-Akt, Akt, p-PI3K, PI3K, VEGFR2, VEGF and GAPDH of EJ and T24 cells treated with 4 μM of cisplatin, 5 mM of matrine and 1 μM of cisplatin and 2 mM of matrine in combination (**a**, **f**) for 48 h. Histograms present the relative grey value of the related proteins measured by ImageJ (**b**–**e**, **g**–**j**). All data are shown as the mean ± SD of three independent experiments. *P < 0.05, **P < 0.01, ***P < 0.001, or ****P < 0.0001 versus the control group

To explore the relevant signal pathway, we performed western blotting to measure the expression levels of Bax, Bcl-2, Cleaved Caspase-3, Caspase-3, p-Akt, Akt, p-PI3K, PI3K, VEGFR2 and VEGF in EJ and T24 cells which were treated by matrine, cisplatin alone and in combination. Results showed the expressions of Bax and Cleaved Caspase-3 were up-regulated in all the treatment groups, but the expressions of Bcl-2, Caspase-3, p-Akt, p-PI3K, VEGFR2, and VEGF protein were down-regulated, while the total Akt, PI3K, and GAPDH levels remained the same (Fig. 7). The effect of combination treatment showed the most significant difference compared with single drug treatment.

### Binding modes of matrine and cisplatin in the tested proteins

**Fig. 8:**
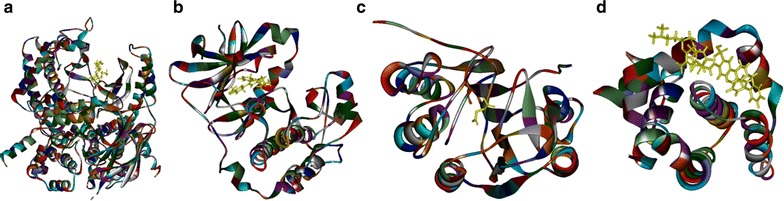
The 3D structures of crystal structures of the tested proteins. The 3D crystal structure of human PI3K with an endogenous ligand (PDB-ID: 4J6I) (**a**), Akt2 with an endogenous ligand (PDB-ID: 2JDR) (**b**), Caspase-3 with an endogenous ligand (PDB-ID: 2XYH) (**c**) and Bcl-2 with an endogenous ligand (PDB-ID: 4IEH) (**d**)

The 3D crystal structures of tested proteins were presented in Fig. 8, all the selected structures were with an endogenous ligand. As shown in Table 2, the RMSD of PI3K, Akt2, Caspase-3, Bcl2 were 0.917, 0.695, 2.111, and 1.370 Å, respectively, which meant that the CDOCKER module in DS 2.5 had a higher accuracy than that of Libdock for the operation. Thus, CDOCKER module was picked as the docking method in the study.

**Table 2:** The validation of molecular docking algorithm (RMSD)

Protein	CDOCKER RMSD (Å)	Libdock RMSD (Å)
(PI3K)4J6I	0.917	3.790
(AKT2)2JDR	0.695	^a^
(Caspase-3)2XYH	2.111	2.546
(Bcl-2)4IEH	1.370	8.190

^a^In Libdock module, both matrine and cisplatin cannot dock with the targeted protein

**Fig. 9:**
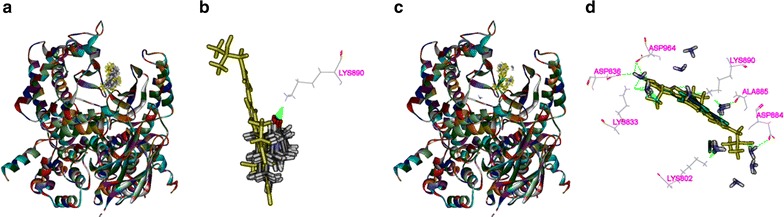
The interactions between the drugs and PI3K(4J6I) in the binding site. Ten random poses of matrine docked into the active site of 4J6I (**a**). The binding modes of matrine in PI3K: at least one residue involved in the interactions in ten random poses, Lys890 (h-bonds) (**b**). Ten random poses of cisplatin docked into the same active site of 4J6I (**c**). The binding modes of cisplatin in PI3K: at least eight residues involved in the interactions in ten random poses, Lys802, Lys833, Asp836, Asp884 Ala885, Lys890 and Asp964 (h-bonds), and Tyr867 (π–π interaction) (**d**)

**Fig. 10:**
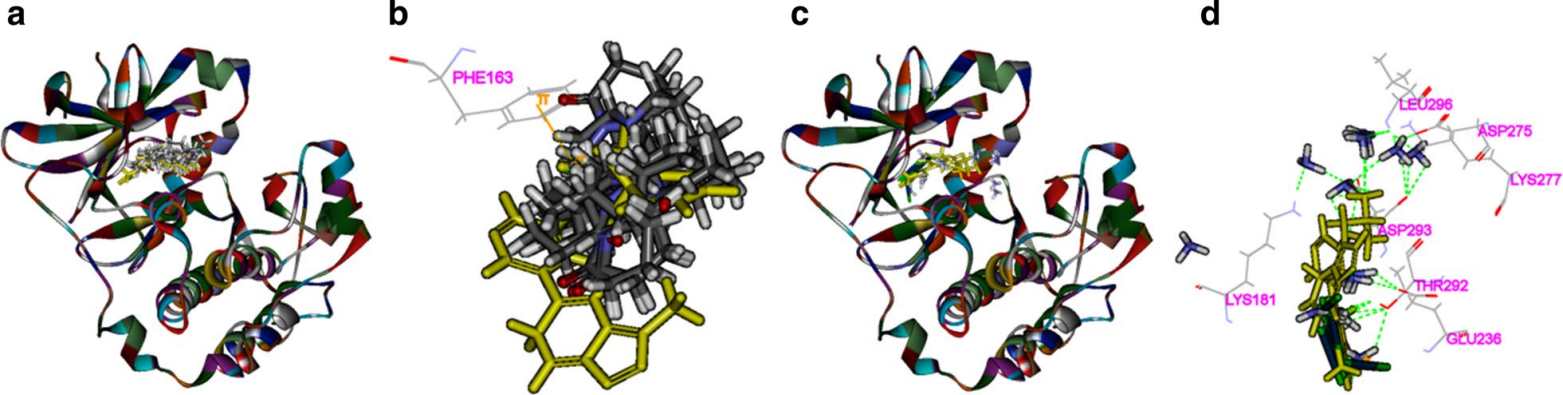
The interactions between the drugs and AKT2(2JDR) in the binding site. Ten random poses of matrine docked into the active site of 2JDR (**a**). The binding modes of matrine in AKT2: at least one residue involved in the interaction in ten random poses, π–π interaction with Phe163 (**b**). Ten random poses of cisplatin docked into the same active site of AKT2 (**c**). The binding modes of cisplatin in AKT2: at least seven residues involved in the interactions in ten random poses, Lys181, Glu236, Asp275, Lys277, Thr292, Asp293, and Leu296 (h-bonds) (**d**)

As shown in Fig. 9, both matrine and cisplatin could be docked into the active site of PI3K in the binding pocket (Fig. 9a, c). Matrine could form H-bonds with Lys890 in the site within PI3K in ten random poses (Fig. 9b), while cisplatin formed H-bonds with Lys802, Lys833, Asp836, Asp884 Ala885, Lys890, Asp964, and π–π interaction with 4J6I by Tyr867 (Fig. 9d). Similarly, both matrine and cisplatin could be docked into the active site of AKT2 in the binding pocket (Fig. 10a, c). Matrine could form π–π interaction with Phe163 in the site within AKT2 in ten random poses (Fig. 10b), while cisplatin formed H-bonds with Lys181, Glu236, Asp275, Lys277, Thr292, Asp293, and Leu296 (Fig. 10d).

**Fig. 11:**
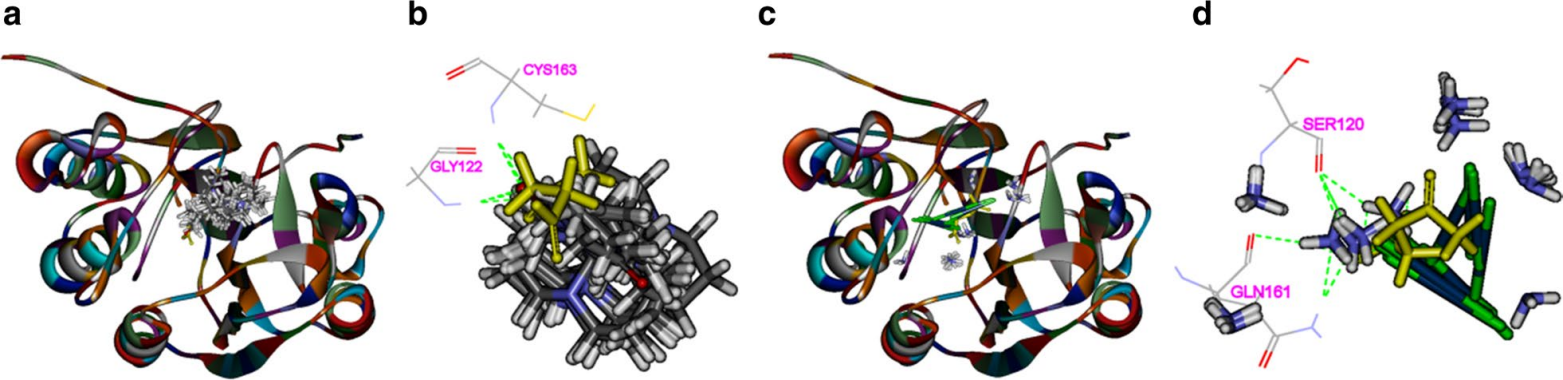
The interactions between the drugs and Caspase-3(2XYH) in the binding site. Ten random poses of matrine docked into the active site of 2XYH (**a**). The binding modes of matrine in Caspase-3: at least two residues involved in the interactions in ten random poses, Gly122 and Cys163 (h-bonds) (**b**). Ten random poses of cisplatin docked into the same active site of 2XYH (**c**). The binding modes of cisplatin in Caspase-3: at least two residues involved in the interactions in ten random poses, Ser120 and Gln161 (h-bonds) (**d**)

**Fig. 12:**
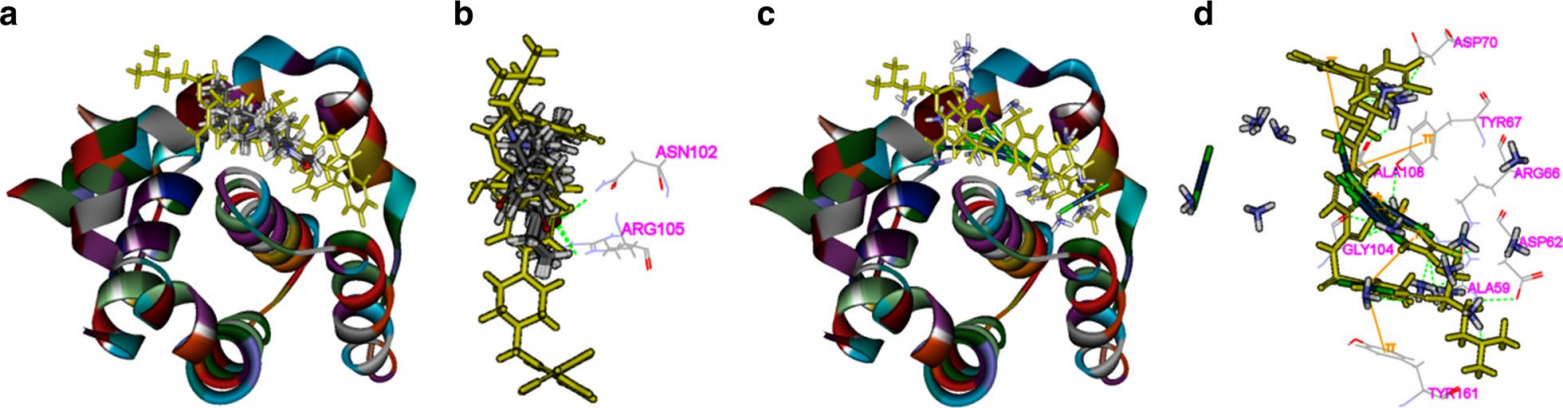
The interactions between the drugs and Bcl-2(4IEH) in the binding site. Ten random poses of matrine docked into the active site of 4IEH (**a**).The binding modes of matrine in Bcl-2: at least two residues involved in the interactions in ten random poses, Asn102, Arg105 (h-bonds) (**b**). Ten random poses of cisplatin docked into the same active site of 4IEH (**c**). The binding modes of cisplatin in 4IEH: at least eight residues involved in the interactions in ten random poses, Ala59, Asp62, Arg66, Try67, Asp70, Gly104 and Ala108 (h-bonds), and Tyr67, Tyr161 (π–π interaction) (**d**)

As shown in Fig. 11, both matrine and cisplatin could be docked into the active site of Caspase-3 in the binding pocket (Fig. 11a, c). Matrine could form π–π interaction with Gly122, Cys163 in the site within Caspase-3 in ten random poses (Fig. 11b), while cisplatin formed H-bonds Ser120, Gln161 (Fig. 11d). Similarly, both matrine and cisplatin could be docked into the active site of Bcl-2 in the binding pocket (Fig. 12a, c). Matrine could form H-bonds with Asn102 and Arg105 in the site within Bcl-2 (Fig. 12b), while cisplatin formed H-bonds with Ala59, Asp62, Arg66, Try67, Asp70, Gly104, Ala108, and π–π interactions with Bcl-2 by Tyr67, Tyr161 (Fig. 12d).

## Discussion

Although herbal medicines have been used for thousands of years in many countries, few components from herbs have been applied on the oncotherapy as single dosage because of their weakness of anti-proliferation. In this study, we found that both matrine and cisplatin had inhibitory effect on the proliferation of the UBC cell lines in a dose- and time-dependent manner, but the inhibitory effect of matrine was much weaker than that of cisplatin. Our further studies showed that the combination of matrine with cisplatin could inhibit cell proliferation, weaken cell repair motility and invasive ability, induce cell cycle arrest, increase the generation of ROS and induce apoptosis in EJ and T24 cells in a synergistic way. Finally, we also revealed that the potential pro-apoptotic mechanisms of matrine and cisplatin on EJ and T24 cells might restrain the VEGF/PI3K/Akt pathway.

Thus, our research indicated that matrine might improve the sensibility to cisplatin for UBC patients while weakening side effects through minimizing the dose of cisplatin.

It is reported that the migration and invasion of cells were decreased after the matrine treatment in human pancreatic cancer and castration-resistant prostate cancer [14, 15]. Our findings showed that both matrine and cisplatin could inhibit the migration and invasion ability of UBC cells, but the combinative treatment exerted more significant inhibitory effect. Furthermore, the expression of EMT related genes could also be influenced by the combinative usage, which indicated that the combination might decrease the UBC cell invasiveness through inhibiting EMT.

More and more studies have shown that the proportion of G1 cells were significantly increased following the treatment of matrine in A549 cells [6], HepG2cell [16], human rhabdomyosarcoma cells [17] and osteosarcoma cells [18]. In our study, we found that matrine could also arrest the cell cycle of EJ and T24 cells at G1 phase, while cisplatin increased primarily the S phase cell percentage in comparison with the untreated cells, which was consistent with many other researchers’ findings [19]. However, Bi’s research found that in gallbladder cancer a proportion of cells at the G1 phase increased compared with that of the control when treated with cisplatin [20]. The effect of cisplatin on the cell cycle distribution is still controversial, and remains to be further explored. Furthermore, the combinative treatment exerted more significant cell cycle arrest at S phase than that of simple cisplatin treatment.

One recent study showed that matrine increased the generation of ROS in a dose- and time-dependent manner, and then increased apoptosis rate in NSCLC cells [21], other studies also found that cisplatin enhanced the generation of ROS in cervical cancer HeLa cells [22]. Our findings revealed that both matrine and cisplatin could produce the same results in EJ and T24 cells, and the combination of them increased the generation of ROS and enhanced apoptosis rate more significantly compared with that of single drug treatment. Taken together, these results suggested that the combination of matrine with cisplatin might increase the generation of ROS, and then promote apoptosis synergistically.

Vascular endothelial growth factor (VEGF) is a major target for the inhibition of tumor vascularisation and tumour growth [23, 24]. The VEGF receptor family in mammals contains three members, VEGFR1, VEGFR2 and VEGFR3 [25]. Notably, VEGFR2 system is a dominant signal-transduction pathway in regulating tumor angiogenesis, specific inhibitors of this pathway inhibit metastases and tumor-cell proliferation, induce apoptosis in tumor cells [26, 27]. For the reason that VEGFR2 improves tumor angiogenesis immediately, it is a proper target for inhibition of solid tumor growth [28]. Therefore, blockage of VEGF/VEGFR2 signaling is the first anti-angiogenic strategy for cancer therapy [29]. The downstream targets of VEGFR2, such as the PI3K/Akt pathway, is one of the most important carcinogenic pathways in many kinds of human cancers [30, 31], which is vital to tumorigenesis, proliferation, invasion, cell cycle progression, inhibition of apoptosis, angiogenesis, metastasis and chemoresistance in cancer cells [30, 32–35]. In our study, both matrine and cisplatin obviously enhanced expressions of Cleaved-Caspase-3 and Bax, while reduced expressions of VEGF, VEGFR2, Bcl-2, Caspase-3, p-PI3K and p-Akt proteins, and total PI3K and Akt proteins expression remained nearly unchanged. The effects of the matrine combination with OXA showed more significant difference compared with those of single drug treatment. Therefore, we considered that matrine and cisplatin induced apoptotic process was likely through down-regulating the VEGF/PI3K/Akt signaling pathway.

Molecular docking, a virtual platform optimized in our previous studies, could be applied to detect the interactions between drug and protein for the underlying mechanisms [13, 36–39]. Our study found that both matrine and cisplatin could be docked into the active pockets of the tested proteins, including PI3K, AKT2, Caspase-3 and Bcl-2. However, even in the same active sites, they still presented different binding modes within ten random poses, which hinted that matrine and cisplatin might interact with the tested proteins in different manners. It could be concluded that the interactions inside the targeted signal pathway might be the underlying molecular mechanisms for the synergy.

To provide further evidence to allow in-depth understanding of the probable mechanisms of the combination of matrine and cisplatin, further studies should be carried out, for example, the synergistic anti-cancer effect of the combination between matrine and cisplatin in vivo should be also verified.

## Conclusions

Our findings demonstrated that combination of matrine with cisplatin could synergistically inhibit the UBC cells through down-regulating VEGF/PI3K/Akt signaling pathway. In the combination, matrine could improve the sensibility of the UBC cell to cisplatin, reduce the dosage of cisplatin, thereby potentially weakening its side effects, indicating that matrine may serve as a new option in the combinative therapy in the treatment of UBC.

## Abbreviations

UBC: urothelial bladder cancer
ROS: reactive oxygen species
FCM: flow cytometry
EMT: epithelial–mesenchymal transition
CID: compound ID
CI: combination index
Fa: fraction affected
PI: propidium iodide
PVDF: polyvinylidenedifluoride
HRP: horseradish peroxidase
ECL: electrochemiluminescence system
2D: two dimensional
RMSD: root mean square deviation
VEGF: vascular endothelial growth factor
